# Reduction of HbA1c levels by fucoxanthin-enriched akamoku oil possibly involves the thrifty allele of uncoupling protein 1 (*UCP1*): a randomised controlled trial in normal-weight and obese Japanese adults

**DOI:** 10.1017/jns.2017.1

**Published:** 2017-02-14

**Authors:** Nana Mikami, Masashi Hosokawa, Kazuo Miyashita, Hitoshi Sohma, Yoichi M. Ito, Yasuo Kokai

**Affiliations:** 1 Department of Biomedical Engineering, Sapporo Medical University School of Medicine, S1W17, Chuo-ku, Sapporo, Hokkaido, Japan; 2 Faculty of Fisheries Sciences, Hokkaido University, Minato-cho 3-1-1, Hakodate, Hokkaido, Japan; 3 Department of Educational Development, Sapporo Medical University Center for Medical Education, S1W17, Chuo-ku, Sapporo, Hokkaido, Japan; 4 Department of Biostatistics, Hokkaido University Graduate School of Medicine, N15W7, Kita-ku, Sapporo, Hokkaido, Japan

**Keywords:** *UCP1*-3826A/G polymorphism, Fucoxanthin, HbA1c, Thrifty gene, Japanese, Diabetes, Glucose metabolism, *β2AR*, *β*2-adrenoreceptor gene, *β3AR*, *β*3-adrenoreceptor gene, Fx, fucoxanthin, FxOH, fucoxanthinol, REE, resting energy expenditure, *UCP1*, uncoupling protein 1

## Abstract

Lifestyle-related problems are becoming a major health threat in East Asian countries. Therefore, finding an efficacious nutraceutical for this population is important. One candidate is fucoxanthin (Fx), a carotenoid abundantly found in edible brown seaweed that has been associated with a number of valuable health-promoting benefits. Unfortunately, clinical studies of Fx are limited. In the present study, we aimed to evaluate the effects of Fx on obesity-related parameters in Japanese subjects harbouring an SNP associated with lifestyle-related problems. In all, sixty normal-weight and obese Japanese adults with BMI over 22 kg/m^2^ were single-blinded and randomly assigned to three Fx-dose cohorts and administered Fx-enriched akamoku oil containing Fx at 0, 1 or 2 mg/d for 8 weeks (*n* 20 per group). Parameters relating to obesity and serum Fx metabolites were measured before and after intervention, but no significant differences were observed between and within the groups. Despite no changes in visceral fat areas and resting energy expenditures after intervention, we observed a significant decline in HbA1c levels in the 2 mg/d Fx group compared with that in the 0 mg/d group (*P* < 0·05), which was correlated with an increase in serum fucoxanthinol (Fx metabolite) levels. In addition, HbA1c levels declined more significantly in subjects with G/G alleles of the uncoupling protein 1 (*UCP1*) gene than in those with the A/A and A/G alleles (*P* < 0·05). We conclude that although Fx supplementation does not affect visceral fat areas, it may reduce HbA1c levels in those harbouring the thrifty allele of *UCP1*-3826A/G.

Fucoxanthin (Fx) is a marine carotenoid widely found in edible brown algae such as wakame (*Undaria pinnatifida*), kombu (*Laminaria japonica*) and akamoku (*Sargassum horneri*). It constitutes more than 10 % of naturally produced carotenoids^(^^1^^)^, providing an abundant reserve of unutilised resources in the ocean. Out of the fifteen species of brown algae that have been studied, akamoku contains the highest content of Fx^(^^2^^)^, making it an excellent source for the raw material in our study.

Fx has demonstrated beneficial effects against obesity^(^^3^^,^^4^^)^, diabetes^(^^5^^)^ and cancer^(^^6^^)^. However, despite proven effects in animal studies, only a few studies on kinetics^(^^7^^,^^8^^)^ and obesity^(^^9^^)^ have been conducted in human subjects. There is one report demonstrating that the supplement Xanthigen^®^, a product that contains Fx and pomegranate seed oil, induces weight loss and body fat reduction, and increases resting energy expenditure (REE) in obese women^(^^9^^)^. However, that study was based on the Caucasian population in Russia; thus, the supplement's effects on East Asian populations remain unclear. Furthermore, no reports have evaluated Fx absorption, serum metabolite (fucoxanthinol; FxOH) levels and functionality. Our study will help expand the knowledge base in these areas.

Although inactive, food-satiated lifestyles dramatically increase the incidence of obesity and related metabolic disorders, the interaction between environmental and genetic factors greatly influences health and susceptibility to disease^(^^10^^)^. Therefore, a tailored nutritional approach based on the individual and the population's genetic background, as characterised by SNP analysis, is required to consider the effectiveness of nutraceuticals.

Uncoupling protein 1 (UCP1) is a thermogenic protein that dissipates glucose and lipid as heat. We investigated how Fx affects glucose metabolism with an SNP of the *UCP1* gene (−3826A/G)^(^^11^^)^, the thrifty G/G genotype, which is associated with the development of obesity and insulin resistance^(^^12^^)^. In addition, we analysed polymorphisms of other thrifty genes, β2-adrenoreceptor (*β2AR*) and β3-adrenoreceptor (*β3AR*), which also decrease energy expenditure^(^^13^^,^^14^^)^.

Through our research, we aimed to elucidate the role of Fx in the development of metabolic disorders such as obesity and diabetes, and study the effect of genetic variation in the Japanese adult population on its function.

## Materials and methods

### Materials

Fx capsules were purchased from Kaneka Corporation. Fx-enriched akamoku oil (containing 1 % Fx) derived from akamoku extract, and medium-chain TAG oil containing lecithin and vitamin E were used to manufacture the Fx soft-gel capsules. Each capsule contained Fx-enriched akamoku oil at the doses of 0 mg (Fx 0 mg, hereafter), 110 mg (Fx 1 mg, hereafter), or 220 mg (Fx 2 mg, hereafter) (Table 1). All capsules appeared identical.

**Table 1.** Composition of fucoxanthin capsules

	Fucoxanthin content		
Composition	0 mg	1 mg	2 mg
Fucoxanthin-enriched akamoku oil (1 % fucoxanthin; mg)	0	110	220
Medium-chain TAG oil (mg)	250	140	30

### Subjects

A total of sixty normal-weight and obese Japanese adult men and women with BMI ≥ 22 kg/m^2^ were included in the study in Rumoi, Hokkaido, Japan. The group consisted of twenty men and forty women who ranged in age from 30 to 77 years. Written informed consent was obtained from all subjects. Pregnant and lactating women and those unable to give consent were excluded from the study.

### Study design

This study was an 8-week, single-centre, randomised, single-blinded, controlled clinical trial conducted from November 2013 to March 2014.

Following simple randomisation procedures (computerised random numbers)^(^^15^^)^, our subjects were single-blinded and randomly assigned to three treatment groups (allocation ratio, 1:1:1): Fx 0 mg/d (placebo control), Fx 1 mg/d or Fx 2 mg/d. The subjects were told to take one capsule per d after dinner for 8 weeks and maintain their normal diet and physical activity.

This study was conducted according to the guidelines of the Declaration of Helsinki, and our study protocol and all procedures involving human subjects were approved by the local research ethics committee in Rumoi Cohortopia (#13-01) and registered with the University Hospital Medical Information Network Clinical Trials Registry (trial number UMIN000020119; http://www.umin.ac.jp/). This report follows the Consolidated Standards of Reporting Trials (CONSORT) 2010 checklist (http://www.consort-statement.org/).

### Visceral fat analyses

Before and after the 8-week supplementation period, abdominal visceral fat areas (primary outcome of this study) were measured in a supine position via computed tomography scans using an Aquilion 64 (Toshiba Medical Systems Corporation) at the Rumoi City Hospital (Hokkaido, Japan).

### Resting energy expenditure measurements

Before and after the 8-week supplementation period, REE was determined with a MedGem metabolic analyser device (HealtheTech, Inc.), a portable indirect calorimeter that we used for 10 min (as recommended by the manufacturer) to measure O_2_ consumption^(^^16^^)^.

### Blood biochemical markers

Before and after the 8-week supplementation period, blood samples were drawn after 12 h of fasting to assess secondary outcomes. The following levels were measured: blood glucose, HbA1c, serum insulin, total cholesterol, HDL-cholesterol, LDL-cholesterol, TAG, aspartate aminotransferase, alanine aminotransferase and γ-glutamyl transpeptidase. Glycated albumin levels were measured before and after intervention only in *UCP1* G/G carriers who were administered Fx 2 mg/d.

While blood glucose is the amount of glucose present in blood at any given time, glycated albumin and HbA1c (glycated Hb) reflect the cumulative glycaemic history (average glycaemic levels over the previous 2 weeks and 3–4 months, respectively)^(^^17^^)^. Serum insulin is an indicator of insulin secretion in a sample population. Total cholesterol, HDL-cholesterol, LDL-cholesterol and TAG reflect lipid metabolism, whereas HDL- and LDL-cholesterol, and TAG levels are used for diagnosing dyslipidaemia. Aspartate aminotransferase, alanine aminotransferase and γ-glutamyl transpeptidase are enzymes related to liver function and are used to evaluate the hepatic toxicity of Fx.

### Serum fucoxanthinol analysis

To quantify serum FxOH levels (a major metabolite of Fx), serum epoxyxanthophyll fractions that included FxOH were extracted and analysed by a liquid chromatography–tandem MS (LC-MS/MS) system as previously developed by the authors and described below. We used Fx as the internal standard because Fx is structurally similar to FxOH^(^^18^^)^, the two have comparable solvent properties, and a preliminary experiment revealed that Fx is not detectable in the serum after its intake.

The extraction was performed according to the method developed by Asai *et al*.^(^^8^^)^, with a slight modification. In brief, 1 ml of serum, 0·2 ml of saline and 2 ml of methanol that included 5 ng of Fx as an internal standard were added to a glass tube and vortexed. The residue was dissolved in *n*-hexane–diethyl ether (9:1, v/v) and applied to a Bond Elut ALN (100 mg, 1 ml) solid-phase extraction cartridge (Agilent Technologies) pretreated with 1 ml *n*-hexane. Epoxyxanthophylls eluted with 1 ml diethyl ether–ethanol (4:1, v/v) were dried and re-suspended in 250 µl of methanol–acetonitrile (70:30, v/v), and then 50 µl of the sample were subjected to LC-MS/MS analysis. Each serum sample was independently extracted and analysed in triplicate.

Our liquid chromatography system was composed of an Agilent 1100 series degasser, binary pump, auto-sampler, column oven (Agilent Technologies) and Inertsil^®^-ODS column (4·6 mm inner diameter × 150 mm, 3 µm, GL Sciences Inc.). The HPLC mobile phases were solvent A (methanol–acetonitrile, 70:30, v/v) and solvent B (dichloromethane). Elution after sample injection was performed as follows: 0–5 min, 0 % B; 5–25 min, 0–100 % B; 25–50 min, 100 % B; 50–60 min, 0 % B. The flow rate was maintained at 1 ml/min at 35°C. FxOH was detected at 2·3 min. The amount of FxOH in the serum was calculated from the peak area counts of the spiked internal standard.

### Analysis of UCP1 gene polymorphism

For analysis of SNP, genomic DNA was isolated from whole-blood samples of each subject using a DNA solution kit (Qiagen). Polymorphisms of *UCP1*-3826A/G (rs1800592), *β2AR* 16 Arg/Gly (rs1042713) and *β3AR* 64 Trp/Arg (rs4994) were determined using a Toyobo Gene analysis system (Fukui) as described by Yamada *et al*.^(^^19^^)^.

### Statistics

The sample size (*n* 20 per group) was calculated from a previous study reporting a 10 % reduction of body weight for obese subjects^(^^9^^)^. Twenty participants were estimated to be sufficient to set the power of the trial at 80 % with a significance level of 5 % for detecting significant body-weight reduction. That number allowed for a 20 % dropout rate from each group.

Results are expressed as means and standard errors. Data were verified for normality by the Shapiro–Wilk test. When necessary, they were log-transformed for normal distribution before further statistical analysis for homogeneity of variance. Parametric comparisons were performed using *t* tests, one-way ANOVA and the Tukey–Kramer test for multiple comparisons. If normal distribution and equality of variance tests were not performed, Welch's test was used, and, if necessary, a non-parametric Steel–Dwass test. For the genotype frequency of *UCP1*-3826A/G, Fisher's exact test was performed. For G/G carriers administered Fx 2 mg/d, albumin levels were measured before and after intervention, then analysed by paired *t* test. In all cases, *P* values < 0·05 were considered statistically significant. All analyses were performed using JMP^®^ 10.0.0 and 10.0.2 (SAS Institute Inc.) or R version 3.2.0.

## Results

### Characteristics of subjects

**Fig. 1.**
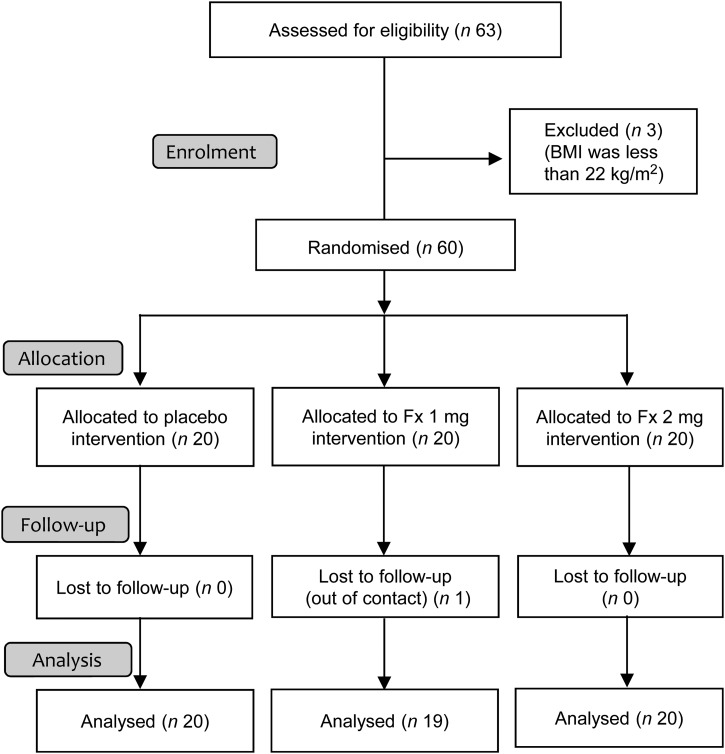
Flow diagram of Japanese adult subjects enrolled in the fucoxanthin (Fx) clinical trial. Subjects were randomised, allocated to Fx intervention groups (0, 1 or 2 mg/d) and followed up for 8 weeks.

This study was a single-blinded, randomised intervention trial. A total of sixty healthy participants provided informed consent regarding the aims of this clinical trial. Allocation, follow-up and analysis are summarised in Fig. 1.

At baseline, the subjects were evenly distributed in terms of age, sex, BMI, visceral fat area, REE and all blood biochemical markers (Table 2). Overall, the mean age was 55 (se 2) years (range 30–77 years) and the mean BMI was 26·4 (se 0·4) kg/m^2^ (range 22·4–34·9 kg/m^2^). Because the Japan Society for the Study of Obesity defines 18·5 ≦ BMI < 25 kg/m^2^ as normal weight and BMI ≧ 25 kg/m^2^ as obese^(^^20^^)^, our population included twenty-six normal-weight and thirty-three obese subjects. One man in the Fx 1 mg/d group dropped out for private reasons. The follow-up rate was 98·3 %, and no adverse events were reported. After intervention, no significant differences for any of the parameters were observed among the three groups (Table 2). Furthermore, comparisons within groups (baseline *v.* after intervention) also revealed no significant differences (Table 2).

**Table 2.** Obesity-related parameters for Japanese adults in the fucoxanthin (Fx) intervention groups (0 or 1 or 2 mg/d) at baseline and after intervention* (Mean values with their standard errors)

	Baseline							After							Baseline *v.* after		
	Fx 0 mg		Fx 1 mg		Fx 2 mg			Fx 0 mg		Fx 1 mg		Fx 2 mg			Within group (*P*)		
	Mean	se	Mean	se	Mean	se	*P*	Mean	se	Mean	se	Mean	se	*P*	Fx 0 mg	Fx 1 mg	Fx 2 mg
Subjects							0·24										
Number	20		19		20												
Male %	20		32		45												
Age (years)	53·0	2·2	57·5	2·9	55·2	3·2	0·52										
Height (cm)	157·6	1·7	158·2	1·8	159·9	2·4	0·68										
Weight (kg)	62·8	2·0	67·0	3·3	70·2	3·3	0·23	64·2	2·5	64·6	3·4	70·1	3·4	0·28	0·90	0·90	0·87
BMI (kg/m^2^)	25·2	0·5	26·6	0·9	27·2	0·7	0·06	25·1	0·5	26·3	0·9	26·9	0·7	0·17	0·95	0·79	0·82
Visceral fat area (cm^2^)	91·6	9·0	119·4	17·4	113·5	8·3	0·24	85·0	7·7	110·8	14·6	107·8	7·8	0·09	0·58	0·80	0·62
Resting energy expenditure (kcal/d)†	1342	58	1326	76	1467	108	0·54	1400	52	1383	65	1561	97	0·18	0·46	0·58	0·42
Blood biochemistry																	
Glucose (mg/dl)†	109	7	107	3	111	3	0·19	106	7	108	5	105	3	0·26	0·06	1·00	0·13
HbA1c (%)	5·4	0·3	5·2	0·1	5·2	0·1	0·61	5·4	0·2	5·2	0·1	5·1	0·1	0·33	0·28	0·94	0·32
Insulin (μU/ml)	8·2	0·7	8·3	0·8	8·8	1·1	0·98	7·0	0·7	7·9	0·8	8·3	0·9	0·51	0·16	0·69	0·86
Total cholesterol (mg/dl)†	207	8	210	9	205	10	0·93	216	9	216	9	205	9	0·54	0·43	0·66	0·94
HDL-cholesterol (mg/dl)†	59	2	57	2	57	3	0·80	60	3	57	2	60	3	0·82	0·99	0·93	0·59
LDL-cholesterol (mg/dl)†	121	8	118	7	122	8	0·95	130	8	129	8	119	9	0·59	0·45	0·31	0·84
TAG (mg/dl)†	129	15	177	46	132	24	0·23	132	17	130	15	141	28	0·77	0·97	0·81	0·84
Aspartate aminotransferase (IU)	21	2	21	1	24	2	0·56	21	2	22	2	24	2	0·79	0·86	0·72	0·62
Alanine aminotransferase (IU)	21	2	23	2	30	5	0·31	21	2	23	3	28	5	0·55	0·95	0·81	0·78
γ-Glutamyl transpeptidase (IU)	47	18	33	6	44	11	0·77	43	13	32	5	46	14	0·90	0·77	0·86	1·00

* Subjects were randomly assigned to three groups. Comparisons of parameters among the three groups (baseline and after) and within a group (baseline *v.* after) were performed by *t* test, one-way ANOVA, Welch's test and a non-parametric Steel–Dwass test. No significant differences were observed between and within the three groups.

† To convert kcal to kJ, multiply by 4·184; to convert glucose from mg/dl to mmol/l, multiply by 0·0555; to convert cholesterol from mg/dl to mmol/l, multiply by 0·0259; to convert TAG from mg/dl to mmol/l, multiply by 0·0113.

### Fucoxanthin did not change obesity-related outcomes

Fx intervention did not change visceral fat areas (change in the Fx 0, 1 and 2 mg/d groups were −6·6 (se 3·6), −8·6 (se 5·4) and −5·7 (se 3·4) cm^2^, respectively). Other obesity-related outcomes such as body weight, BMI and REE also remained unchanged in the Fx groups.

### Fucoxanthin reduced HbA1c and increased serum fucoxanthin metabolite levels

Detailed analysis of biochemical parameters revealed a significant reduction in HbA1c in the Fx 2 mg/d group (−0·14 (se 0·0) %) compared with that of the 0 mg/d group (0·06 (se 0·05) %, *P* < 0·05; Table 3). This was associated with an apparent increase in serum FxOH (*P* < 0·0001), a metabolite of Fx (Table 3). Indeed, significant increases in serum FxOH levels detected between the 1 mg/d and 0 mg/d groups (*P* < 0·0001), and between the 2 mg/d and 0 mg/d groups (*P* < 0·0001) indicated a dose-dependent effect (Table 3), with mean FxOH levels of 2·7 and 2·1 and 0·4 nm, for the 2, 1 and 0 mg/d groups, respectively.

**Table 3.** Changes in HbA1c levels and serum fucoxanthinol (FxOH) levels in the fucoxanthin (Fx) intervention groups (0 or 1 or 2 mg/d) after the 8-week intervention† (Mean values with their standard errors)

	Fx 0 mg		Fx 1 mg		Fx 2 mg	
	Mean	se	Mean	se	Mean	se
ΔHbA1c (%)	0·06	0·05	0·01	0·05	−0·14*	0·05
Serum FxOH levels (after 8 weeks) (nm)	0·4	0·0	2·1**	0·2	2·7**	0·4

Mean value was significantly different from that of the 0 mg/d group: * *P* < 0·05, ** *P* < 0·0001.

† ΔHbA1c represents the change in HbA1c levels before and after an 8-week intervention of Fx. *P* values for comparison of parameters among the three groups were calculated by one-way ANOVA, the Tukey–Kramer test for multiple comparisons and a non-parametric Steel–Dwass test.

### Possible interaction between fucoxanthin supplementation and UCP1-3826A/G polymorphism may influence reduction of HbA1c

**Fig. 2.**
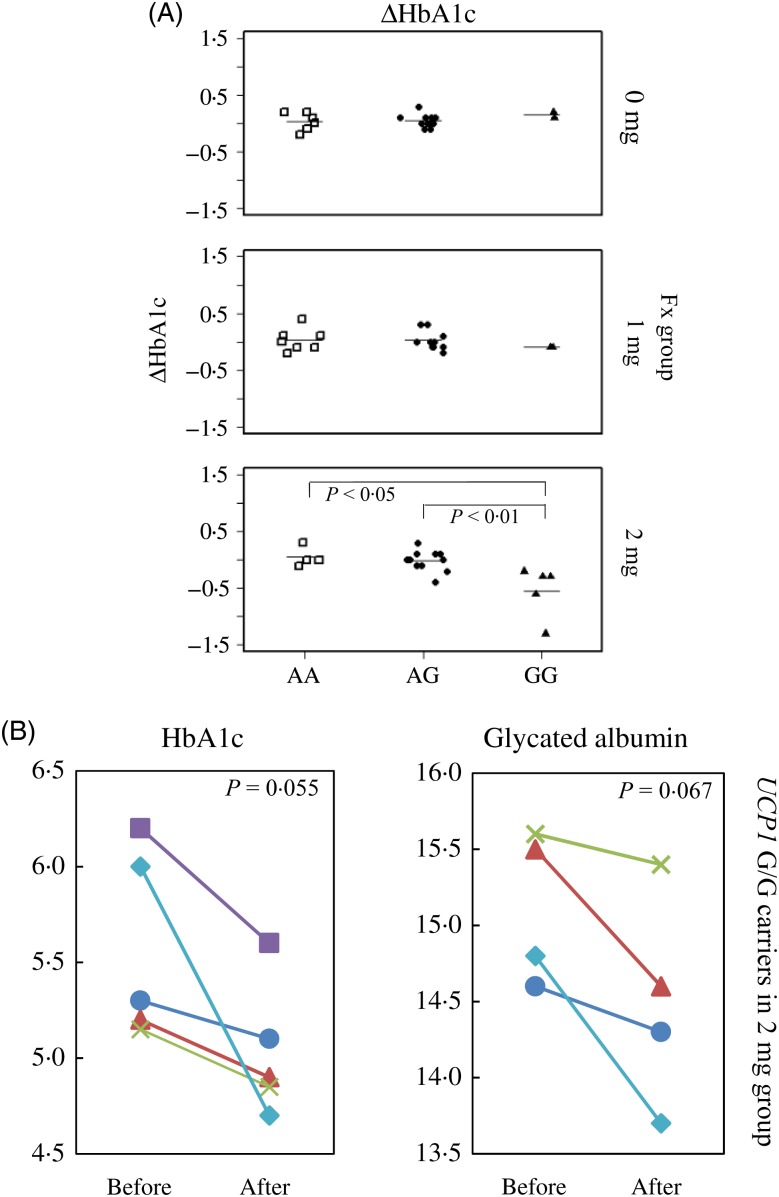
Reduction in HbA1c levels after 8 weeks of fucoxanthin (Fx) treatment with possible involvement of the thrifty allele of uncoupling protein 1 (*UCP1*) in Japanese adults. (A) ΔHbA1c with *UCP1*-3826A/G genotype. In the 2 mg/d group: A/A *v.* G/G (*P* < 0·05) and A/G *v.* G/G (*P* < 0·01). (B) Changes in HbA1c levels and glycated albumin before and after the 8-week intervention in G/G genotype carriers of the 2 mg/d group (*n* 5). Both HbA1c and glycated albumin levels declined for all G/G genotype carriers in the 2 mg/d group. Paired *t* tests for comparisons of the changes in HbA1c and glycated albumin levels (before and after intervention) yielded *P* = 0·055 and *P* = 0·067, respectively. Glycated albumin levels from one subject could not be determined because of a lack of sample volume.

**Fig. 3.**
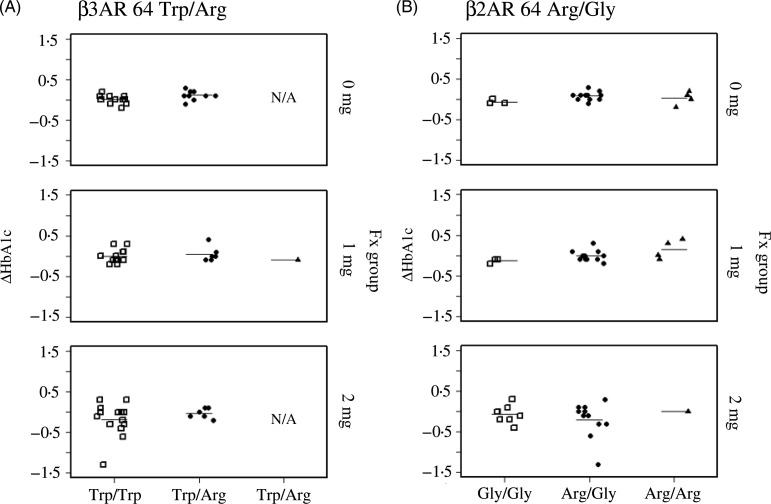
Effects of other well-known thrifty genotypes on 8-week fucoxanthin (Fx)-induced changes in HbA1c levels in Japanese adults. ΔHbA1c in subjects with (A) *β*3-adrenoreceptor (*β3AR*) 64 Trp/Arg and (B) *β*2-adrenoreceptor (*β2AR*) Arg/Gly polymorphisms. We were unable to observe the effect of these two polymorphisms on Fx-induced reduction of HbA1c levels because of the limited number of carriers with each genotype. N/A, not applicable.

As shown in Fig. 2(A), 8 weeks of supplementation with 2 mg/d of Fx significantly reduced HbA1c levels in subjects with the *UCP1*-3826A/G polymorphism. The reduction in HbA1c levels for the G/G genotype (−0·54 (se 0·45) %) was significantly greater than that of the A/A (0·05 (se 0·17) %, *P* < 0·05) and A/G (−0·02 (se 0·18) %, *P* < 0·01) genotypes. We noted that the sample number for each *UCP1*-3826A/G genotype was not significantly different among Fx groups (*P* = 0·63, Fisher's exact test), and that HbA1c levels did not decline in the Fx 0 or 1 mg/d groups (Fig. 2(A)). Additionally, serum FxOH levels were not affected by the *UCP1* genotype (Supplementary Fig. S1). Parallel plot analysis confirmed that Fx intervention reduced HbA1c and glycated albumin levels in all Fx 2 mg/d participants who carried the *UCP1* G/G allele. *P* values from the paired *t* test for changes in HbA1c and glycated albumin (before intervention *v.* after intervention) were 0·055 and 0·067, respectively (Fig. 2(B)). We could not analyse the involvement of other well-known thrifty genes, such as *β3AR* 64 Trp/Arg and *β2AR* Arg/Gly, with Fx-induced reduction of HbA1c because of the lack of carriers for each genotype (Fig. 3).

### Baseline parameters were unbiased according to UCP1 genotype

Table 4 shows the baseline characteristics of each *UCP1* genotype group. At baseline, the participants of each genotype were evenly distributed in terms of age, sex, BMI, visceral fat area, REE and all blood biochemical markers.

**Table 4.** Baseline characteristics of trial subjects stratified into three genotypes of uncoupling protein 1 (*UCP1*)-3826A/G polymorphism in Japanese adults* (Mean values with their standard errors)

*UCP1*-3826A/G genotype…	A/A		A/G		G/G		
	Mean	se	Mean	se	Mean	se	*P*
Subjects							
Number	17		33		9		0·54
Male %	24		33		44		
Age (years)	55·1	3·2	54·7	2·2	57·1	3·7	0·88
Weight (kg)	66·0	3·9	66·6	2·1	68·2	4·0	0·86
BMI (kg/m^2^)	25·6	0·8	26·8	0·6	25·9	0·7	0·48
Visceral fat area (cm^2^)	88·6	8·8	112·2	10·8	129·2	13·5	0·14
Resting energy expenditure (kcal/d)†	1388	113	1340	57	1504	107	0·43
Blood biochemistry							
Glucose (mg/dl)†	112	8	108	2	109	4	0·57
HbA1c (%)	5·4	0·3	5·2	0·1	5·4	0·2	0·29
Insulin (μU/ml)	7·1	0·9	9·0	0·7	8·6	1·4	0·18
Total cholesterol (mg/dl)†	209	10	211	7	191	13	0·39
HDL-cholesterol (mg/dl)†	58	3	58	2	57	3	0·92
LDL-cholesterol (mg/dl)†	120	10	123	5	111	14	0·46
TAG (mg/dl)†	142	20	152	29	127	25	0·83
Aspartate aminotransferase (IU)	20	2	21	1	28	4	0·08
Alanine aminotransferase (IU)	24	5	22	2	33	7	0·19
γ-Glutamyl transpeptidase (IU)	36	7	40	11	57	23	0·38

* Subjects were stratified into three genotypes of *UCP1. P* values for comparison of parameters among the three genotypes were calculated using one-way ANOVA, Welch's test and a non-parametric Steel–Dwass test. No significant differences were found.

† To convert kcal to kJ, multiply by 4·184; to convert glucose from mg/dl to mmol/l, multiply by 0·0555; to convert cholesterol from mg/dl to mmol/l, multiply by 0·0259; to convert TAG from mg/dl to mmol/l, multiply by 0·0113.

### Safety

In the present study, Fx capsules were well tolerated by all participants who completed the 8-week trial. No subjective or objective adverse effects, including hepatic dysfunction and alterations in lipid metabolism, were reported.

## Discussion

Our present study showed that intake of Fx-enriched akamoku oil significantly increased serum FxOH levels in a fashion of Fx dose-dependency. The increase was associated with HbA1c reduction, remarkably in carriers with the G/G allele of the *UCP1* gene. These results suggest that Fx intake affects HbA1c reduction in UCP1 polymorphism. Further studies are needed to elucidate a mechanistic link between Fx intake and glucose metabolism in G/G allele carriers.

Genetic variations generate innate characteristics in individuals and the population, as shown by genome-wide association studies^(^^21^^)^. Because of the great diversity and complexity of genetic backgrounds, tailoring healthcare through the use of rational genotyping such as SNP analysis is rising in importance. For instance, the thrifty *UCP1* G/G genotype is one of the most well-characterised genes associated with the development of obesity and insulin resistance^(^^12^^)^. It is conceivable that carriers of the G/G allele, comprising over 20 % of the East Asian population^(^^22^^,^^23^^)^, could be at high risk for obesity-related disorders. It is also possible that carriers of the G/G allele may respond to treatment differently from the general population. For that reason, we aimed to elucidate the biofunctional effects of Fx on metabolic disorders in the Japanese population.

Our study was a single-blinded, randomised controlled clinical trial. In all, sixty participants were assigned to three groups (*n* 20 per group): 0, 1 or 2 mg/d of Fx per d for 8 weeks. Contrary to a previous report in mice^(^^24^^)^, no adverse effects involving serum biochemical markers of the liver and lipid dysfunction were reported.

In a previous study, 8 weeks of supplementation with Xanthigen^®^ (containing Fx and pomegranate seed oil) decreased the body weight of Russian women^(^^9^^)^. However, those results were not reproduced in our study. Indeed, we detected no changes in obesity-related outcomes, including visceral fat area, which could be explained by the many differences between the two study populations (e.g. race, sex, age, body weight and BMI). Further, the reduced body weight in Russian women could have been related to the combined effects of Xanthigen^®^ including Fx and pomegranate seed oil.

Orally administered Fx is hydrolysed to FxOH in the intestinal tract, after which it is absorbed^(^^18^^)^ and detected in the blood and organs^(^^25^^)^. We noted a dose-dependent increase in serum FxOH that confirmed oral absorption of Fx. Combined with the reduction of HbA1c for the 2 mg/d group (but not the 0 mg/d group), our results strongly suggest that Fx possesses biofunctionality.

Furthermore, we determined that HbA1c reduction was closely linked to the G/G allele of the *UCP1* gene. Participants in the 2 mg/d group who carried the *UCP1*-3826A/G gene polymorphism exhibited a more appreciable reduction in HbA1c levels. HbA1c, glycated Hb that reflects average blood glucose levels, is considered the standard for assessing glucose metabolism over the long term. Additionally, serum levels of glycated albumin (a product of non-enzymic glycation that is also used as a glycaemic indicator) apparently decreased in all subjects in the 2 mg/d group with the G/G genotype. Although an accurate HbA1c measurement is limited by various conditions, including erythrocyte lifespan, normal ageing and other factors^(^^26^^)^, the decline in both HbA1c and glycated albumin levels suggests that Fx may aid in glucose metabolism. Taken together, our findings indicate a novel role for Fx: *UCP1* genotype-dependent reduction of HbA1c levels in humans, although the mechanism by which Fx exerts this effect remains unclear.

There are other reports supporting a relationship between Fx and *UCP1* gene products: *UCP1* mRNA expression levels in human intraperitoneal adipose tissue are lower in *UCP1* G/G genotype carriers than in the other two genotypes^(^^27^^)^; and Fx induces *UCP1* mRNA and protein expression in murine adipocytes^(^^3^^)^. To expand upon our findings on the relationship between Fx and the *UCP1* genotype, further studies with more participants are needed. In addition, various formulations of Fx or FxOH might be investigated in addition to Fx oil, such as processed algal materials, food matrix-destruction, mixing with lysoglyceroglycolipids^(^^28^^)^, and/or addition of egg yolk to FxOH^(^^29^^)^.

Our data indicate that Fx-induced reduction of HbA1c levels is closely linked to the G/G allele of the *UCP1* gene. Fx's dependence on *UCP1* gene polymorphism sheds light on a possible tailored application for this unique carotenoid. Indeed, tailoring a nutraceutical like Fx would be a rational and efficient treatment strategy for genetically high-risk populations.

## Supplementary material

For supplementary material accompanying this paper visit http://dx.doi.org/10.1017/jns.2017.1. click here to view supplementary material
