# Supplementary figures and images for: Reduction of HbA1c levels by fucoxanthin-enriched akamoku oil possibly involves the thrifty allele of uncoupling protein 1 (UCP1): a randomised controlled trial in normal-weight and obese Japanese adults
Source: J Nutr Sci. 2017 Feb 14;6:e5. doi: 10.1017/jns.2017.1 (PMC5465861; doi:10.1017/jns.2017.1)

## Supplementary Fig.1

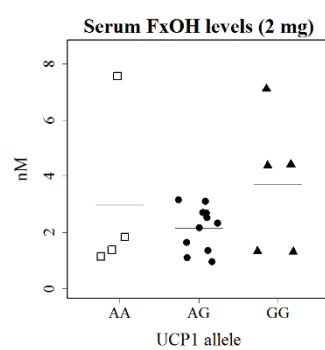

Supplement: Supplementary file 1 [file jnssup.zip › S2048679017000015sup002.pdf]
