# Supplementary material for: Reduction of HbA1c levels by fucoxanthin-enriched akamoku oil possibly involves the thrifty allele of uncoupling protein 1 (UCP1): a randomised controlled trial in normal-weight and obese Japanese adults
Source: J Nutr Sci. 2017 Feb 14;6:e5. doi: 10.1017/jns.2017.1 (PMC5465861; doi:10.1017/jns.2017.1)
Supplement: Supplementary file 1 [file jnssup.zip › S2048679017000015sup001.doc]

**Supplementary Fig. S1.** Serum fucoxanthinol (FxOH) levels after 8-wk intervention in 2-mg/day group with *UCP1* -3826A/G genotype.

There were no significant differences in serum FxOH levels among the three genotypes in the 2-mg/day group.
